# Correlates of Meeting the Muscle-Strengthening Exercise Guidelines in Children and Adolescent

**DOI:** 10.3389/fpubh.2022.854100

**Published:** 2022-05-27

**Authors:** Jiayi Gu, Jin-Tao Hong, Youliang Lin, Jin Yan, Sitong Chen

**Affiliations:** ^1^School of Physical Education and Sport, Shanghai Normal University, Shanghai, China; ^2^Shanghai Research Institute of Sports Science (Shanghai Anti-Doping Agency), Shanghai, China; ^3^Department of Physical Education, Wuhan University of Technology, Wuhan, China; ^4^Centre for Active Living and Learning, University of Newcastle, Callaghan, NSW, Australia; ^5^College of Human and Social Futures, University of Newcastle, Callaghan, NSW, Australia; ^6^Institute for Health and Sport, Victoria University, Melbourne, VIC, Australia

**Keywords:** physical activity, promotion, muscle-strengthening exercise, school-aged children factors, China

## Abstract

This study aimed to explore the potential correlates of muscle-strengthening exercise (MSE) in Chinese children and adolescents. A convenient sample (*n* = 3733) was recruited into this study. Self-reported questionnaires were used to collect information on sex, grade, ethnicity, residence, family composition, moderate to vigorous physical activity (MVPA), family income, parent's education level and MSE in children and adolescents as well as their parent(s). The prevalence of meeting the MSE guidelines was 62.1%. Children and adolescents who were in primary or middle school were more likely to meet the MSE guidelines ([primary school] OR = 2.33, 95% CI: 1.16–4.68; [middle school] OR = 4.62, 95% CI: 2.27–9.39). Children and adolescents with Han ethnicity had a higher likelihood to meet the MSE guidelines (OR = 1.97, 95% CI: 1.37–2.83). Children and adolescents meeting the MVPA recommendation were more likely to meet the MSE guidelines (OR = 5.41, 95% CI: 3.97–7.37). Relative to those who had a parent not meeting the MSE guidelines, those with either father or mother meeting the MSE guidelines were more likely to meet the MSE guidelines (OR = 1.32, 95% CI: 1.13–1.55). Our study may offer evidence for future MSE interventions in Chinese children and adolescents.

## Introduction

As a neglected form of health-enhancing physical activity (PA), muscle-strengthening exercise (MSE) epidemiological research is still limited (1), even though the World Health Organization (WHO) recommend that children and adolescents as well adults should engage in MSE (e.g., barbells or dumbbells) a week (2) because of its numerous health benefits, including bone health promotion (3), muscular fitness improvement (3), obesity prevention (4) and mental disorders discouragements (5, 6). At present, however, the relevant studies on MSE focus on the adult population instead of the young population (1). For example, some national surveys (e.g., the USA, Australia, Scotland) merely reported the estimated prevalence of meeting the age-specific MSE recommendation in adults (at least 2 times a week) (2) and these studies indicated that adults had insufficient participation in MSE (3, 7, 8), especially on western adult populations (9). Despite this, a recent systematic review demonstrated that the relationship between MSE and sedentary behaviors, sleep during childhood is still needed more evidence to support (10).

Across the previously published literature, sufficient attention has been laid on correlates of MSE in adult populations rather than other young populations. Specifically, Smith and his colleagues conducted many national surveys to report the levels of MSE and then explore its correlates in adult populations from various countries (11). For example, in German adults, 58.3% of them reported no MSE (5). Among 9345 Australian participants, the prevalence of meeting the MSE guidelines was 18.6 (95% CI: 17.5–19.7), and it is associated correlates included sex, age and self-rated health (8). In the US, the nationally representative data showed that 30.2% of American adults met the MSE guidelines while 57.8% of them had no MSE at all per week (3). This study also suggested that sex, income, education level, obesity and aerobic activity engagements, as well as self-rated health, were significant correlates of MSE (3). Recently, some researchers who are not from western countries have begun to pay attention to this research topic. Specifically, using a sample of Chinese adults from Hubei Province, Lin and Yan (9) found that the prevalence of meeting the MSE guidelines was 28.5%, and its correlates were sex and family composition (single or multiple kids). Based on those studies, it is acknowledged that there is sufficient evidence base to understand the adult population's engagements in MSE.

However, to our knowledge, so far, little is known about the levels and correlates of MSE among children and adolescents (7). Across a limited number of studies, some researchers merely reported the levels and explore the correlates of MSE, respectively, among western countries' children and adolescents, like Australia (11), the US (12) and Canada (13). For example, a recently published study by Smith et al. (11) found that only two factors, including resisting training efficacy and moderate to vigorous physical activity, were associated with MSE in a sample of Australian adolescents (*n* = 602). For the US children and adolescents, boys were more likely to report meeting the MSE guidelines than were girls (adjusted OR = 1.65, 95% CI: 1.43–1.91) (12). In another study consisting of the US samples (Los Angeles Unified School District), the authors also indicated sex is an important correlate of meeting the MSE guidelines (14). In Canada, the health survey data showed that only 53.7% of 45,298 grade 9–12 students reported having more than 3 times of MSE per week (13). The reported levels of MSE and associated factors with MSE across the literature may help researchers better understand insufficient engagements of MSE in children and adolescents, which can, in turn, help design effective interventions aiming at promoting MSE. However, so far, little is known about the MSE status focused on Chinese young people, while a majority of physical activity studies focused on the overall physical activity instead of specific components of physical activity (15, 16). This is a barrier for capturing true levels of MSE and its potential correlates, which provides insights into a comprehensive understanding of physical activity in Chinese young people. The current evidence gaps would be negative to design and implement MSE interventions or promotions. In addition to MSE interventions, recent research has stressed the importance of promotiong physical literacy in children and adolescents. A physically literure individual should engage in sufficient physical activity behaviors, including MSE (17). From the perspective of physical literacy promotion, it is needed to examine which factors would be associated with the components of physical literacy.

To better understand MSE among Chinese children and adolescents, evidence of levels and correlates of the MSE based on empirical studies is required. In this regard, it is recommended that Chinese researchers should explore the correlates of MSE among Chinese children and adolescents. Addressing the evidence gaps in the literature needs a great deal of empirical research. The objectives of the present study were, therefore, to report levels of MSE in a sample of Chinese children and adolescents and its potential correlates (e.g., intrapersonal, interpersonal and behavioral aspects).

## Methods

### Study Design and Participants

The study design and methodology has been reported elsewhere (9). In brief, the present study was a cross-sectional aiming to explore parents' impacts on their kids' (children and adolescents) healthy active lifestyles, conducted from May to June 2019 in the Hubei Province of China. Using convenience sampling, three public schools (one primary school, one middle school, and one high school) were invited from each city (13 cities in the Hubei Province; 39 schools were in total invited) to participate in the survey. Finally, 3733 study participants (age range: 10-19 years) and one of their parents (either mother or father) provided valid information for all variables pertaining to this study. The study protocol and procedure were approved by the Institutional Review Board (IRB) of Wuhan University of Technology in March 2019. Participants and their legal guardians provided written consent. To enhance the reliability and validity of the survey, the research staff promised that participants' information will be strictly protected and treated anonymously (only use for research aims).

## Measurements

### Study Outcome (Muscle-Strengthening Exercise, [MSE])

Study participants were asked to report their information on MSE. The question was that “how many times did you do MSE designed to strengthen your muscles last week?”. In this context, MSE was defined as “activities to be done involving major muscle groups, like a push-up, weightlifting, curl-up or pull-up” to help participants better understand and fill in the questionnaire easily. Response times for participants were 0-7 days. This measure has been considered reliable and valid in assessing young people's MSE (12, 18). Based on the well-recognized MSE guidelines (World Health Organization, 2020), in this study, the variable of MSE was treated as a binary variable in the statistical analyses (0 = not meet [reporting 0-2 days], 1 = meet [reporting 3-7 days]).

### Study Exposures

Each study participant was asked to report the following information. For intrapersonal information, participants reported their sex (boy or girl), grade (4, 5, 6…12), China's ethnic groups (Han or minority), residence (urban or rural), family composition (single children or multiple children), For behavioral information, each study participants reported that how many days engaging in moderate to vigorous physical activity (MVPA) via the Chinese version of the Health Behavior in School-aged Children (HBSC) questionnaire which has been validated in Chinese (19), and this measure is a well-recognized questionnaire that has been used widely in China's physical activity epidemiological research (20–22). The following item was used: How many days did you engage in MVPA at least 60 min on weekdays over the past week? (0 = none, 1 = 1 day, 2 = 2 days, 3 = 3 days, 4 = 4 days, 5 = 5 days, 6 = 6 days, and 7 = 7 days). Study participants reporting 7 days were regarded as meeting the MVPA guidelines (2, 23). Study participants' parent reported their family income and educational level using a self-reported questionnaire designed for parents (either father or mother). Parent-reported their family income (personal annual income in a family) (<9000; 9001–30,000; 30,001–100,000; >100,000; [Unit: Chinese Yuan]). The same question of assessing children and adolescents' MSE was also used to collect information on parents' MSE. For adults, parent reporting at least 2 days was regarded as meeting the MSE guidelines (2).

### Statistical Analyses

First, descriptive statistics were used to report the prevalence of meeting the MSE guidelines (met vs. not met) and exposure variables (sex: male or female; grade group: primary school, middle school or high school; ethnicity: Han or Minority; residence: urban or rural; family composition: single or two or more children; parent's education level: primary school or below, middle school, high school, occupation school, graduate, postgraduate or above; personal income: <9,000 or 9,000–30,000 or 30,000–100,000 or >100,000; children's MVPA guidelines: not meeting or meeting; parent's MSE guidelines: not meeting or meeting; children's MSE guidelines: not meeting or meeting). Second, *Spearman's rho* coefficients were calculated to determine the bivariate associations among study variables (exposures and outcome). Third, to assess the associations between selected factors (study exposure) and whether or not meeting the MSE guidelines, multilevel logistic regression (also called hierarchical regression) was used. This technique takes into account the dependency of observations within a or multiplies clusters (in this case, individual characteristics were nested within schools, and schools were nested within districts). A multilevel logistic regression controls for confounding at the micro-level (individual), but in addition controls for confounding at the macro-level (schools or districts). Based on our data structure of this study, we fitted our data as a three-level structure (level 3: district; level 2: school; level 1: individual), which then uses a multilevel logistic regression analysis with Restricted Maximum Likelihood Estimation (REML) was conducted to examine the associations of MSE with exposure variables. Adjusted odds ratios (ORs) with corresponding 95% confidence intervals (95% CI) were described. Statistical analyses were performed using IBM SPSS 24.0 software (using the Generalized Linear Mixed Models module).

## Results

Table 1 shows the sample characteristics of this study. In total, 3733 children and adolescents were included in the analysis (62.3% boys). The percentages of participants from primary schools, middle schools and high schools were 35.0, 30.65, and 34.4%, respectively. Most of the children and adolescents were of Han ethnicity (74.7%), living in an urban area (80.1%) with no siblings (75.5%). Approximately 30.0% of their parents had received university or above education level. And about two-fifths of participants reported family income between 30,000 to 100,000. Less than 20% of children and adolescents met the MVPA guidelines, while 62.1% of them met the age-specific MSE guidelines with gender differences (*p* < 0.00). As for their parents, 45.7% of parents complied with parents MSE guidelines.

**Table 1:** Sample characteristics of this study.

		**Total**		**Boy**		**Girl**		***p***
		***n***	**%**	***n***	**%**	***n***	**%**	
Total		3733	100	2324	62.3	1409	37.7	*/*
Grade group								
	Primary school	1308	35.0	839	36.1	469	33.3	0.00
	Middle school	1141	30.6	742	31.9	399	28.3	
	High school	1284	34.4	743	32.0	541	38.4	
Ethnicity								
	Han	2788	74.7	1416	60.9	1372	97.4	0.00
	Minority	945	25.3	908	39.1	37	2.6	
Residence								
	Urban	2989	80.1	1900	81.8	1089	77.3	0.00
	Rural	744	19.9	424	18.2	320	22.7	
Siblings								
	Single	2819	75.5	1767	76.0	1052	74.7	0.35
	Two or more	914	24.5	557	24.0	357	25.3	
Parent's education level*								
	Primary school or below	102	2.7	67	2.9	35	2.5	0.00
	Middle school	736	19.7	425	18.3	311	22.1	
	High school	915	24.5	544	23.4	371	26.3	
	Occupation school	870	23.3	539	23.2	331	23.5	
	Graduate	903	24.2	595	25.6	308	21.9	
	Postgraduate or above	207	5.5	154	6.6	53	3.8	
Personal annual income#								
	<9,000	614	16.4	369	15.9	245	17.4	0.02
	9,000-30,000	1030	27.6	610	26.2	420	29.8	
	30,000–100,000	1536	41.1	980	42.2	556	39.5	
	>100,000	553	14.8	365	15.7	188	13.3	
Children's MVPA guidelines								
	Not meeting	3051	81.7	1885	81.1	1166	82.8	0.21
	Meeting	682	18.3	439	18.9	243	17.2	
Parent's MSE guidelines*								
	Not meeting	2028	54.3	1296	55.8	732	52.0	0.02
	Meeting	1705	45.7	1028	44.2	677	48.0	
Children's MSE guidelines								
	Not meeting	1413	37.9	924	39.8	489	34.7	0.00
	Meeting	2320	62.1	1400	60.2	920	65.3	

*either father or mother.

*# annual income per person (Unit as Chinese Yuan)*.

*MSE, muscle-strengthening exercise*.

*MSE guidelines: children and adolescents should amass 3 times per week; adults should amass 2 times per week*.

*MVPA, moderate to vigorous physical activity*.

The bivariate correlations among study variables are shown in Table 2. To be specific, children's MSE guidelines was positively correlated with sex (*r* = 0.051, *p* < 0.01), residence (*r* = 0.012, *p* < 0.01), siblings (*r* = 0.062, *p* < 0.01), children's MVPA guidelines parent's (*r* = 0.289, *p* < 0.01), MSE guidelines (*r* = 0.107, *p* < 0.01), respectively. More information can be found in Table 2.

**Table 2:** Bivariate correlations among study variables.

	**1**	**2**	**3**	**4**	**5**	**6**	**7**	**8**	**9**	**10**
1 Sex	1									
2 Grade group	0.054**	1								
3 Ethnicity	−0.406**	−0.151**	1							
4 Residence	0.054**	−0.195**	−0.132**	1						
5 Siblings	0.015	−0.240**	0.015	0.207**	1					
6 Parent's education level	−0.072**	0.136**	0.128**	−0.362**	−0.248**	1				
7 Personal annual income#	−0.048**	0.063**	0.071**	−0.164**	−0.148**	0.313**	1			
8 Children's MVPA guidelines	−0.021	−0.120**	−0.047**	−0.054**	0.040*	0.030	0.029	1		
9 Parent's MSE guidelines	0.037*	−0.046**	−0.043**	0.020	0.064**	−0.026	−0.041*	0.073**	1	
10 Children's MSE guidelines	0.051**	−0.186**	−0.084**	0.012**	.062**	−0.010	−0.008	0.289**	0.107**	1

*either father or mother.

*# annual income per person (Unit as Chinese Yuan)*.

*MSE, muscle-strengthening exercise*.

*MSE guidelines: children and adolescents should amass 3 times per week; adults should amass 2 times per week*.

*MVPA, moderate to vigorous physical activity*.

*denotes p < 0.05.

**denotes p < 0.01.

Table 3 shows the results by a multilevel logistic regression, revealing the associations between the potential correlates with meeting the MSE guidelines in the study samples. In detail, compared with those who were in high school, children and adolescents who were in primary school and middle school were more likely to meet the MSE guidelines ([primary school] OR = 2.33, 95% CI: 1.16–4.68, *p* = 0.02; [middle school] OR = 4.62, 95% CI: 2.27–9.39. *p* = 0.00). Children and adolescents with Han ethnicity had a higher likelihood to meet the MSE guidelines (OR = 1.97, 95% CI: 1.37–2.83, *p* = 0.00). Children and adolescents living urban areas were more likely to meet the MSE guidelines (OR = 1.26, 95% CI: 1.06–1.70, *p* = 0.01). Regarding MVPA in children and adolescents, the regression results display that children and adolescents meeting the MVPA recommendation was five times greater than those not meeting the MVPA recommendation to meet the MSE guidelines (OR = 5.41, 95% CI: 3.97–7.37, *p* = 0.00). Relative to those who had parents (either father or mother) not meeting the MSE guidelines, children and adolescents with either father or mother meeting the MSE guidelines were more likely to meet the MSE guidelines (OR = 1.32, 95% CI: 1.13–1.55, *p* = 0.00).

**Table 3:** The association between the potential correlates with meeting the MSE guidelines.

		**Beta**	**aOR**	**95% CI**		***p***	
***Intercept (Fixed effects)***	0.97	2.64	1.03	6.75	**0.04**	
Sex						
	Boy	Ref				
	Girl	0.13	1.14	0.96	1.36	0.15
Grade group						
	Primary school	0.85	2.33	1.16	4.68	**0.02**
	Middle school	1.53	4.62	2.27	9.39	**0.00**
	High school	Ref				
Ethnicity						
	Han	0.68	1.97	1.37	2.83	**0.00**
	Minority	Ref				
Residence						
	Urban	0.23	1.26	1.06	1.70	**0.01**
	Rural	Ref				
Family composition						
	Single children	−0.04	0.97	0.79	1.18	0.73
	Two or more	Ref				
Parent's education*						
	Primary school or below	−0.01	0.99	0.53	1.83	0.97
	Middle school	−0.15	0.86	0.57	1.30	0.48
	High school	−0.12	0.89	0.61	1.31	0.55
	Occupation school	0.03	1.04	0.71	1.52	0.86
	Graduate	0.07	1.07	0.74	1.56	0.71
	Postgraduate or above	Ref				
Family income^#^						
	<9,000	0.14	1.15	0.85	1.55	0.36
	9,000–30,000	0.10	1.10	0.84	1.44	0.49
	30,000–100,000	0.15	1.16	0.90	1.49	0.26
	>100,000	Ref				
Children's MVPA guidelines						
	Meet	1.69	5.41	3.97	7.37	**0.00**
	Not meet	Ref				
MSE in parent*						
	Meet	0.28	1.32	1.13	1.55	**0.00**
	Not meet	Ref				
Random and residual effects			Beta	Std Error	*z*	
District variance (level 3)			0.17	0.22	0.77	0.44
School variance (level 2)			0.82	0.28	2.87	**0.01**
Residual			1.00	**/**		

*aOR denotes adjusted odds ratio*.

*CI, confidence interval*.

*either father or mother.

*^#^annual income per person (Unit as Chinese Yuan)*.

*MSE, muscle-strengthening exercise; MSE guidelines: children and adolescents should amass 3 times per week; adults should amass 2 times per week; MVPA, moderate to vigorous physical activity*.

*Bold fonts denote statistically significant (p < 0.05)*.

## Discussion

The present study aimed to report the prevalence of meeting the MSE guidelines (at least 3 times per week) in Chinese children and adolescents, and then explore its correlates. To our knowledge, this is the first reported study based on samples from the Chinese children and adolescents population. The present study found that the majority of Chinese children and adolescents met the MSE guidelines (prevalence = 62.1%). Besides, this study suggested that grade group (age range: 10–19 years), ethnicity, residence, MVPA in children and adolescents, and parent's (either father or mother) MSE were significant correlates of MSE in Chinese children and adolescents.

There were about 6 out of 10 Chinese children and adolescents who met the well-recommended MSE guidelines. The prevalence of meeting the MSE guidelines in our study is relatively higher than that in a study by Smith, Diallo (11). In Smith et al. (10)'s study, the prevalence of meeting the MSE guidelines was 35.2% (lower than ours: 62.1%). When looking at the prevalence of meeting the MSE guidelines in Canadian (54%) and American youths (51%), our result was still higher than those studies. Possible reasons for the noticeable inconsistencies include different sample characteristics and measurements as well as participants' understanding of muscle-strengthening exercises (18). In addition to the possible reasons, physical education classes may be another consideration. Chinese children and adolescents are required to participate in at least three physical education classes per week, which could provide more opportunities for them to practice muscle-related activities during the physical education classes. This would be an incentive for children and adolescents to report more engagements in MSE compared with other counterparts from western countries. However, this assumption should be tested by future cross-cultural studies. We should admit that using self-reported measures to assess the MSE in children and adolescents may result in an overestimation of MSE, which fails to capture true levels of MSE among children and adolescents. It is needed, therefore, to use the more standardized and more accurate measure to capture the MSE in young people (3, 11). Although it seems that Chinese children and adolescents had better performance in MSE than counterparts from western countries, it is still highly recommended to increase the prevalence of meeting the MSE guidelines in the Chinese young population because of its numerous health benefits (6, 8). When considering promoting physical literacy, encouraging behavior changes would be a possible approach, by which increase physical activity levels (24). As MSE is a kind of physical activity, it would be probably promising that children and adolescents who engage in more MSE regularly may show improved physical literacy.

Consistent with some studies (3), our study found that sex, parental socioeconomic status (e.g., parental education level and family income) were not associated with MSE in children and adolescents. However, although sex was not a correlate of MSE in the final full model, in our study, girls reported a higher prevalence of meeting the MSE guidelines than boys. This finding is contrary to Smith et al. (11) and other studies, which indicated that boys had more engagements in MSE than girls. It is interesting and unexpected that Chinese female children and adolescents reported a higher prevalence of MSE. To our knowledge, it is novel research finding that should be explained in future studies because understanding sex difference in MSE is an important research issue for MSE promotion (11). To our knowledge, the associations of parental education and family income with MSE in children and adolescents are firstly studied by our study. However, unlike the positive associations of parental education and family income with overall physical activity, this study found no association between family income and parents' education level. A possible reason is that Chinese parents with higher parental education levels and more family income do not have sufficient awareness of engagements in MSE or knowledge on health benefits from appropriate MSE (19), which in turn do not encourage or cannot influence their kids' MSE. Moreover, the family composition was not associated with MSE in children and adolescents. To our knowledge, this is the first study to assess the association between family composition and MSE in children and adolescents, and we do not have comparable data with our research findings. It is therefore to further explore whether peer influence can affect MSE in children and adolescents.

In our study, grade group (age range: 10-19 years), residence, ethnicity, MVPA of children and adolescents and parent's MSE were significantly associated with meeting the MSE guidelines. Grade group can be regarded as a proxy measure of age which has been recognized as a determinant of physical activity (25–28). Sufficient evidence has shown an age-related decline in physical activity in children and adolescents in China (22, 29, 30). As a form of physical activity, MSE would be negatively influenced by the increasing age of children and adolescents. Another possible explanation relates to academic activities and loads of Chinese children and adolescents (30, 31). With increasing academic activities and loads, Chinese children and adolescents are exposed to less time and fewer opportunities for physical activities throughout adolescence (20–22, 32), which in turn reduce their engagements in MSE. Future studies should confirm this research finding using a longitudinal study design.

Consistent with previous studies, we found that children and adolescents living in urban residences were more likely to report meeting the MSE guidelines. Such a research finding is also consistent with studies based on adults (3, 7, 9). A possible explanation is that those living in urban areas can more easily access facilities and equipment (e.g., bars) for participation in muscle-related activities, which can increase their engagement of MSE (8, 33). However, this explanation may be fitting with adults, which might not be useful in children and adolescents. Hence, it is needed to answer the differences in MSE of children and adolescents from urban and rural areas. Similarly, ethnicity is another important correlate of MSE in children and adolescents in our study. This finding is consistent with some research findings based on adults (29), which is a novel finding in children and adolescents and cannot be fully explained in this study. In this regard, more studies should be encouraged to explore the mechanism of differences in MSE across children and adolescents with various ethnicities.

Accumulated MVPA is associated with MSE in our study, which is consistent with Smith et al. (11) and Roth et al. (14). Although this study shares concordant findings with previous studies, it must be noted that the measures of MVPA and MSE were very similar. It may be a reason why there was a positive association between MVPA and MSE. However, the measure for MVPA did not distinguish between aerobic physical activity and MSA. Hence, future studies should use objective or device-based measures to assess MVPA, which in turn explores its relationships with MSE more reliably and accurately (26). Another consideration to further answer the association between MVPA and MSE in children and adolescents is the measurement of MSE. It is well-known that the most widely used measures for MSE are self-reported questionnaires (7). The weaknesses of self-reported questionnaires have been reported (3), which is prone to recall measurement bias. Taken together, to provide a more comprehensive insight into the association between MVPA and MSE in children and adolescents, studying how to use objective or device-based measures to monitor MSE is an alternative and necessary approach.

Parent's MSE is a significant correlate related to MSE in children and adolescents. To our knowledge, it is a novel research finding appearing in the literature for the first time, which can advance our knowledge and researchers' understanding of MSE. However, owing to the novelty, there is no comparable data or evidence that can support or negate this important research finding. To explain this research finding, some hypotheses are proposed that need further confirmations or tests. A parent's MSE can be viewed as a role model. It has been well-documented that parental modeling can act as an incentive to promote physical activity in children and adolescents (34, 35). Many studies have shown that parent's participation in physical activity tends to make their kids engage in more physical activity (36–38), because the parent(s) can play a role model in increasing children and adolescents' physical activity level (36). As a form of physical activity, it is expected that parents' MSE is positively associated with MSE in children and adolescents in our study. In addition to this, there is some evidence that shows children and adolescents appear to imitate their parent's behavior (6, 39). In this regard, it is reasonable that children and adolescents with parent(s) who have sufficient MSE tend to engage in more MSE. However, these assumptions based on previous frameworks to explain parents' influences on children and adolescents' MSE should be further examined comprehensively, to advance our understanding of MSE in young people.

Although this study is one of very few to report the level of MSE in Chinese children and adolescents, as well as its potential correlates, this study also presents some limitations. First, the cross-sectional design could not determine causality. Thus, the real causal relationship of some factors with MSE is uncertain. Second, the self-reported measure was used to obtain information on MSE, which caused recall bias and in turn limited the generalization of our research findings to the wider society. Thus, the application of the research findings to the wider population would need to be done with caution. Fourth, owing to measurement limitations, we were not able to capture more information on MSE like duration, type, or location. This resulted in uncertainties about the profiles of MSE among study participants. Future studies should determine more correlates of MSE among Chinese children and adolescents, to expand on the current research and in turn design more effective interventions for improved health promotion effectiveness.

## Conclusion

Approximately 60% of Chinese children and adolescents met the recommended levels of MSE. MSE in Chinese children and adolescents can be associated with grade group, residence, ethnicity, MVPA in children and adolescents, as well as parent's MSE. This study can advance understanding of MSE in China. For public health benefits, large efforts should be made to increase young people's participation in MSE by multiple aspects. From the perspective of public health policymakers, it should be acknowledged that the lack of emphasis on MSE in public health policy is one of the potential culprits for the lack of children and adolescents' engagement in MSE. Therefore, stressing the significance of MSE in public health policy is the priority. Especially, policymakers should speed up the development and implementation of school physical activity or sports policies. Future public MSE interventions should prioritize older and minority children and adolescents, those living in rural areas. Also, parents (s) should play a role model in engaging in more MSE for promoting their kids' MSE.

## Data Availability Statement

The raw data supporting the conclusions of this article will be made available by the authors, without undue reservation.

## Ethics Statement

The studies involving human participants were reviewed and approved by Wuhan University of Technology. Written informed consent to participate in this study was provided by the participants' legal guardian/next of kin.

## Author Contributions

JYG, J-TH, YLL, and JY were involved in the study design and drafted the manuscript. JY and STC performed data analysis. STC and JY were involved in the revision of the manuscript. All authors contributed to the article and approved the submitted version.

## Conflict of Interest

The authors declare that the research was conducted in the absence of any commercial or financial relationships that could be construed as a potential conflict of interest.

## Publisher's Note

All claims expressed in this article are solely those of the authors and do not necessarily represent those of their affiliated organizations, or those of the publisher, the editors and the reviewers. Any product that may be evaluated in this article, or claim that may be made by its manufacturer, is not guaranteed or endorsed by the publisher.

## References

[1] Bennie JA Shakespear-Druery J De Cocker K. Muscle-strengthening exercise epidemiology: a new frontier in chronic disease prevention. Sports Med-Open. (2020). 6:1–8. 10.1186/s40798-020-00271-w 32844333 PMC7447706

[2] Bull FC Al-Ansari SS Biddle S Borodulin K Buman MP Cardon G. World Health Organization 2020 guidelines on physical activity and sedentary behaviour. Br J Sports Med. (2020). 54:1451–62. 10.1136/bjsports-2020-102955 33239350 PMC7719906

[3] Bennie JA Lee DC Khan A Wiesner GH Bauman AE Stamatakis E. Muscle-strengthening exercise among 397,423 US adults: prevalence, correlates, and associations with health conditions. Am J Prev Med. (2018). 55:864–74. 10.1016/j.amepre.2018.07.022 30458949

[4] Bennie JA Pedisic Z van Uffelen JG Charity MJ Harvey JT Banting LK. Pumping iron in Australia: prevalence, trends and sociodemographic correlates of muscle strengthening activity participation from a national sample of 195,926 adults. PLoS ONE. (2016). 11:e0153225. 10.1371/journal.pone.0153225 27119145 PMC4847788

[5] Bennie JA Teychenne M Tittlbach S. Muscle-strengthening exercise and depressive symptom severity among a nationally representative sample of 23,635 german adults. J Affect Disord. (2020). 266:282–7. 10.1016/j.jad.2020.01.172 32056889

[6] De Cocker K Teychenne M White RL Bennie JA. Adherence to aerobic and muscle-strengthening exercise guidelines and associations with psychological distress: a cross-sectional study of 14,050 English adults. Prevent Med. (2020). 139:106192. 10.1016/j.ypmed.2020.106192 32640287

[7] Bennie JA De Cocker K Pavey T Stamatakis E Biddle SJ Ding D. Muscle strengthening, aerobic exercise, and obesity: a pooled analysis of 1.7 million US adults. Obesity. (2020). 28:371–8. 10.1002/oby.22673 31709754

[8] Bennie JA Pedisic Z van Uffelen JG Gale J Banting LK Vergeer I. The descriptive epidemiology of total physical activity, muscle-strengthening exercises and sedentary behaviour among Australian adults–results from the national nutrition and physical activity survey. BMC Pub Health. (2015). 16:1–13. 10.1186/s12889-016-2736-3 26809451 PMC4727339

[9] Lin Y Yan J. Muscle-Strengthening Activities and Sociodemographic Correlates among Adults: Findings from Samples in Mainland China. Int J Environ Res Pub Health. (2020). 17:2266. 10.3390/ijerph17072266 32230937 PMC7177312

[10] Smith JJ Eather N Weaver RG Riley N Beets MW Lubans DR. Behavioral correlates of muscular fitness in children and adolescents: a systematic review. Sports Med. (2019). 49:887–904. 10.1007/s40279-019-01089-7 30864143

[11] Smith JJ Diallo TM Bennie JA Tomkinson GR Lubans DR. Factors associated with adherence to the muscle-strengthening activity guideline among adolescents. Psychol Sport Exe. (2020). 51:101747. 10.1016/j.psychsport.2020.101747

[12] Song M Carroll DD Fulton JE. Meeting the 2008 physical activity guidelines for Americans among US youth. Am J Prevent Med. (2013). 44:216–22. 10.1016/j.amepre.2012.11.016 23415117

[13] Harvey A Faulkner G Giangregorio L Leatherdale ST. An examination of school-and student-level characteristics associated with the likelihood of students' meeting the Canadian physical activity guidelines in the COMPASS study. Canadian J Public Health. (2017). 108:348–54. 10.17269/CJPH.108.5925 29120304 PMC6972312

[14] Roth SE Gill M Chan-Golston AM Rice LN Crespi CM Koniak-Griffin D. Physical activity correlates in middle school adolescents: Perceived benefits and barriers and their determinants. J School Nurs. (2019). 35:348–58. 10.1177/1059840518780300 29895181 PMC6703952

[15] Shen H Yan J Hong JT Clark C Yang XN Liu Y. Prevalence of physical activity and sedentary behavior among chinese children and adolescents: variations, gaps, and recommendations. Int J Environ Res Public Health. (2020). 17:3066. 10.3390/ijerph17093066 32354193 PMC7246713

[16] Ren T Yan J Sun Q. Sociodemographic correlates of organized sports participation in a sample of middle school students in China. Front Public Health. (2021). 9:1655. 10.3389/fpubh.2021.730555 34869148 PMC8636987

[17] Tompsett C Burkett B McKean MR. Development of physical literacy and movement competency: a literature review. J Fit Res. (2014). 131:3. 10.1002/ajim.23295 34523153 PMC8653178

[18] Milton K Varela AR Strain T Cavill N Foster C Mutrie N. A review of global surveillance on the muscle strengthening and balance elements of physical activity recommendations. J Frailty, Sarcopenia Falls. (2018). 3:114. 10.22540/JFSF-03-114 32300699 PMC7155319

[19] Liu Y Wang M Tynjälä J Lv Y Villberg J Zhang Z. Test-retest reliability of selected items of Health Behaviour in School-aged Children (HBSC) survey questionnaire in Beijing, China. BMC Med Res Methodol. (2010). 10:1–9. 10.1186/1471-2288-10-73 20696078 PMC2927607

[20] Chen ST Liu Y Tremblay MS Hong JT Tang Y. Meeting 24-h movement guidelines: Prevalence, correlates, and the relationships with overweight and obesity among Chinese children and adolescents. J Sport Health Sci. (2021). 10:349–59. 10.1016/j.jshs.2020.07.002 32679341 PMC8167320

[21] Chen ST Yan J. Prevalence and selected sociodemographic of movement behaviors in schoolchildren from low-and middle-income families in Nanjing, China: a cross-sectional questionnaire survey. Children. (2020). 7:13. 10.3390/children7020013 32069924 PMC7072474

[22] Chen ST Liu Y Hong JT Tang Y Cao ZB Zhuang J. Co-existence of physical activity and sedentary behavior among children and adolescents in Shanghai, China: do gender and age matter? BMC Public Health. (2018). 18:1–9. 10.1186/s12889-018-6167-1 30466431 PMC6251113

[23] Tremblay MS Carson V Chaput JP Connor Gorber S Dinh T Duggan M. Canadian 24-h movement guidelines for children and youth: an integration of physical activity, sedentary behaviour, and sleep. App Physiol, Nutr Metabol. (2016). 41:S311–27. 10.1139/apnm-2016-0151 27306437

[24] Zhang C Liu Y Xu S Sum RK Ma R Zhong P. Exploring the level of physical fitness on physical activity and physical literacy among Chinese university students: a cross-sectional study. Front Psychol. (2022) 2:900. 10.3389/fpsyg.2022.833461 35369138 PMC8966725

[25] Bauman AE Reis RS Sallis JF Wells JC Loos RJ Martin BW. Correlates of physical activity: why are some people physically active and others not? The Lancet. (2012). 380:258–71. 10.1016/S0140-6736(12)60735-1 22818938

[26] Sallis JF Prochaska JJ Taylor WC. A review of correlates of physical activity of children and adolescents. Med Sci Sports Exe. (2000). 32:963–75. 10.1097/00005768-200005000-00014 10795788

[27] Trost SG Owen N Bauman AE Sallis JF Brown W. Correlates of adults' participation in physical activity: review and update. Med Sci Sports Exe. (2002). 34:1996–2001. 10.1097/00005768-200212000-00020 12471307

[28] Sterdt E Liersch S Walter U. Correlates of physical activity of children and adolescents: a systematic review of reviews. Health Edu J. (2014). 73:72–89. 10.1177/001789691246957820630024

[29] Fan X Cao ZB. Physical activity among Chinese school-aged children: national prevalence estimates from the 2016 physical activity and fitness in China—The youth study. J Sport Health Sci. (2017). 6:388–94. 10.1016/j.jshs.2017.09.006 30356592 PMC6189233

[30] Liu Y Tang Y Cao ZB Zhuang J Zhu Z Wu XP. Results from the China 2018 Report Card on physical activity for children and youth. J Exe Sci Fitness. (2019). 17:3. 10.1016/j.jesf.2018.10.002 30662507 PMC6323175

[31] Zhu Z Tang Y Zhuang J Liu Y Wu X Cai Y. Physical activity, screen viewing time, and overweight/obesity among Chinese children and adolescents: an update from the 2017 physical activity and fitness in China—the youth study. BMC Public Health. (2019). 19:1–8. 10.1186/s12889-019-6515-9 30767780 PMC6376726

[32] Zhu X Haegele JA Tang Y Wu X. Physical activity and sedentary behaviors of urban Chinese children: Grade level prevalence and academic burden associations. BioMed Res Int. (2017) 2017:40147. 10.1155/2017/7540147 29435459 PMC5757099

[33] Freeston J Gale J Mavros Y Bennie JA Pedisic Z Bauman AE Stamatakis E. Associations between multiple indicators of socio-economic status and muscle-strengthening activity participation in a nationally representative population sample of Australian adults. Prevent Med. (2017). 102:44–8. 10.1016/j.ypmed.2017.06.020 28648930

[34] Beets MW Cardinal BJ Alderman BL. Parental social support and the physical activity-related behaviors of youth: a review. Health Educ Behav. (2010). 37:621–44. 10.1177/1090198110363884 20729347

[35] Petersen TL Møller LB Brønd JC Jepsen R Grøntved A Association between parent and child physical activity: a systematic review. Int J Behav Nutr Phy Act. (2020). 17:1–16. 10.1186/s12966-020-00966-z 32423407 PMC7236180

[36] Liu Y Zhang Y Chen S Zhang J Guo Z Chen P. Associations between parental support for physical activity and moderate-to-vigorous physical activity among Chinese school children: a cross-sectional study. J Sport Health Sci. (2017). 6:410–5. 10.1016/j.jshs.2017.09.008 30356620 PMC6189258

[37] Khan SR Uddin R Mandic S Khan A. Parental and peer support are associated with physical activity in adolescents: evidence from 74 countries. International journal of environmental research and public health. (2020). 17:4435. 10.3390/ijerph17124435 32575699 PMC7344886

[38] Reimers AK Schmidt SC Demetriou Y Marzi I Woll A. Woll. Parental and peer support and modelling in relation to domain-specific physical activity participation in boys and girls from Germany. PLoS ONE. (2019). 14:e0223928. 10.1371/journal.pone.0223928 31665192 PMC6821055

[39] Schoeppe S Vandelanotte C Bere E Lien N Verloigne M Kovacs E. The influence of parental modeling on children's physical activity and screen time: does it differ by gender? Eu J Public Health. (2017). 27:152–7. 10.1093/eurpub/ckw182 28177458

